# Electroencephalography-functional magnetic resonance imaging using a familial temporal lobe epilepsy model in cats: an exploratory methodological pilot study

**DOI:** 10.1093/jvimsj/aalag209

**Published:** 2026-09-21

**Authors:** Takayuki Miura, Rikako Asada, Daisuke Hasegawa

**Affiliations:** Laboratory of Veterinary Clinical Neurology, Graduate School of Nippon Veterinary and Life Science University, Musashino, Tokyo 180-8602, Japan; Veterinary Medical Teaching Hospital, Nippon Veterinary and Life Science University, Musashino, Tokyo 180-8602, Japan; Laboratory of Veterinary Clinical Neurology, Graduate School of Nippon Veterinary and Life Science University, Musashino, Tokyo 180-8602, Japan

**Keywords:** electroencephalography, epilepsy, epileptogenic zone, functional magnetic resonance imaging

## Abstract

**Background:**

Reliable methods for identifying the epileptogenic zone (EZ) in veterinary medicine remain limited. Although intracranial electroencephalography (EEG) is considered the most dependable approach for EZ localization, noninvasive simultaneous EEG and functional magnetic resonance imaging (EEG-fMRI) has been applied in human medicine as an alternative.

**Hypothesis/Objectives:**

Develop and evaluate EEG-fMRI methodology for clinical application in veterinary medicine and evaluate the methodological feasibility of EEG-fMRI using cats with familial spontaneous epilepsy (FSECs).

**Animals:**

Ten cats with clear interictal epileptiform discharges on scalp EEG were selected from an FSEC colony with previously suggested amygdalo-hippocampal involvement.

**Methods:**

Each cat underwent 3 scalp EEG and EEG-fMRI sessions. After preprocessing, blood oxygen level-dependent (BOLD) activation maps were generated. Primary analyses evaluated amygdalo-hippocampal activation using small-volume correction (SVC) and group-level analysis.

**Results:**

The BOLD activation was detected in all sessions at an exploratory threshold, but no activation survived correction for multiple comparisons. The SVC analysis identified amygdalo-hippocampal activation in 16/30 sessions. Group-level analysis identified the largest cluster extending from the left parahippocampal gyrus to the hippocampal body.

**Conclusions and clinical importance:**

This exploratory pilot study showed that EEG-fMRI could be technically implemented in FSECs, but individual-level EZ localization remains insufficient for clinical use. Further optimization, data from surgical candidates, and multimodal comparisons are needed.

## Introduction

Epilepsy surgery requires accurate identification of the epileptogenic zone (EZ).^1^ In human medicine, multimodal noninvasive evaluations, including seizure semiology, long-term video electroencephalography (VEEG), neuropsychological testing, structural magnetic resonance imaging (MRI), interictal fluorodeoxyglucose positron emission tomography (FDG-PET), magnetoencephalography, and ictal single-photon emission computed tomography are used. When these findings are inconclusive or deep foci are suspected, invasive electrocorticography (ECoG) or stereotactic electroencephalography (SEEG) is required.^2^^,^^3^

In veterinary medicine, epilepsy surgery has recently emerged.^4–9^ However, reliable EZ localization remains challenging despite advances in surgical techniques. Presurgical evaluations for dogs and cats include neurologic examination, scalp EEG, ictal video-based semiology, long-term VEEG, structural MRI, interictal FDG-PET, and intracranial EEG, but evidence for these modalities remains limited and diagnostic accuracy requires improvement.^10^

Intracranial VEEG monitoring is considered the most reliable method for EZ identification in human medicine, with electrode placement often using navigation systems or robotic guidance.^11^^,^^12^ However, comparable techniques are rarely available in veterinary medicine,^3^ and stereotactic planning is prone to placement error. Although intracranial EEG has been reported in freely moving dogs and cats, its feasibility is constrained by motion artifacts, device-related complications, costs, ethical considerations, and owner acceptance. Moreover, invasive recordings such as ECoG and SEEG sample only restricted brain regions, making accurate electrode placement dependent on precise noninvasive presurgical localization.

Electroencephalography–functional magnetic resonance imaging (EEG-fMRI), introduced in human medicine in the 1990s,^13^^,^^14^ combines the temporal resolution of EEG with the spatial resolution of blood oxygenation level-dependent (BOLD) fMRI. Electroencephalography–functional magnetic resonance imaging can visualize epileptogenic activity from superficial to deep structures. Unlike conventional task-based fMRI, EEG-fMRI treats interictal epileptiform discharges (IEDs) as event-related stimuli and identifies associated BOLD responses. This technique has demonstrated concordance with scalp EEG and intracranial EEG and has shown value as a presurgical tool in humans with epilepsy.^15–18^ To our knowledge, EEG-fMRI has not been reported in dogs or cats. Establishing this modality may improve noninvasive localization, guide invasive electrode placement, and decrease reliance on invasive testing.

Our pilot study aimed to evaluate the methodological feasibility of EEG-fMRI in veterinary medicine. Using cats with familial spontaneous epilepsy (FSECs) with previously suggested amygdalo-hippocampal involvement,^12^^,^^19–21^ we evaluated whether simultaneous EEG-fMRI could be technically implemented using MRI-compatible EEG devices and a clinically applicable immobilization protocol, whether interpretable in-scanner EEG recordings could be obtained after artifact correction, and whether IED-related BOLD activation could be detected within this predefined region.

## Materials and methods

### Animals

Ten FSECs were included based on previous reports indicating that the amygdalo-hippocampal region represents the presumed EZ.^12^^,^^19–21^ Details of seizure history and EEG findings are provided in Table 1 and the Appendix.

**Table 1:** Subject information.

Cat #	Age (Y)	Sex	Body weight (kg)	Seizure frequency	Session #	IED count (out-of-scanner EEG)	Top 3 regions (out-of-scanner EEG)	IED count (in-scanner EEG)	Top 3 regions (in-scanner EEG)
**Cat 1**	4	F	2.7	3/year	1	45 (4.5/min)	LT RC LC	114 (9.5/min)	LO RO LF
					2	75 (7.5/min)	LF LC LT	64 (5.3/min)	LF RF LO
					3	41 (4.1/min)	LF RF LT	86 (7.2/min)	LO LF RO
**Cat 2**	1	M	3.7	1-2/year	1	32 (3.2/min)	LF LT RT	105 (8.75/min)	RO LT RT
				VS+	2	40 (4.0/min)	LC RF LF	73 (6.1/min)	LO LC LT
					3	42 (4.2/min)	LF LC RF	87 (7.3/min)	LO LT RT
**Cat 3**	11	F	3.3	–	1	61 (6.1/min)	LC LF LT	119 (9.9/min)	LO RO LF
					2	41 (4.1/min)	RF LT LF	68 (5.7/min)	LO RO LT
					3	30 (3.0/min)	LF RF LT	74 (6.2/min)	LO RO LF
**Cat 4**	11	M	4.5	<1/year	1	28 (2.8/min)	LT LF RT	127 (10.6/min)	LO RO LT
				VS+	2	24 (2.4/min)	LF LC LT	114 (9.5/min)	LO RO LF
					3	30 (3.0/min)	LC RT LT	112 (9.3/min)	LO RF RO
**Cat 5**	1	F	3.2	–	1	50 (5.0/min)	LF LT RC	102 (8.5/min)	LO LT RF
					2	39 (3.9/min)	LC LT LF	63 (5.3/min)	LO LF LC
					3	44 (4.4/min)	LC LF RF	51 (4.3/min)	LO RF LC
**Cat 6**	2	F	2.1	–	1	64 (6.4/min)	LF LC RT	125 (10.4/min)	LO RF RO
					2	23 (2.3/min)	LC LF RF	56 (4.7/min)	LO RO RF
					3	37 (3.7/min)	LC LF RF	86 (7.2/min)	LF LT LC
**Cat 7**	2	F	2.8	–	1	39 (3.9/min)	LC LF RO	73 (6.1/min)	LO RO RF
					2	54 (5.4/min)	RF RT RC	51 (4.3/min)	LO LT LC
					3	42 (4.2/min)	LF RC LC	67 (5.6/min)	RO LO RF
**Cat 8**	1	M	3.4	–	1	27 (2.7/min)	LC LF LO	80 (6.7/min)	LO RF RO
					2	26 (2.6/min)	LC LF RC	39 (3.3/min)	LO RO LC
					3	13 (1.3/min)	RF LC RC	38 (3.2/min)	RF LO RO
**Cat 9**	12	M	5.3	–	1	48 (4.8/min)	LF LC LT	79 (6.6/min)	RO LO LF
					2	37 (3.7/min)	LC LF LO	64 (5.3/min)	LO RF RO
					3	50 (5.0/min)	LC RC RT	54 (4.5/min)	LO RO LF
**Cat 10**	1	M	4.4	–	1	20 (2.0/min)	LC LF LT	69 (5.8/min)	LO RO LC
					2	27 (2.7/min)	LC LF RO	54 (4.5/min)	RO LO RT
					3	27 (2.7/min)	LC RF RT	45 (3.8/min)	LO LC RO

IED counts are presented as the total number of IEDs, with values in parentheses indicating IED frequency (IEDs per minute). The top 3 regions indicate the 3 regions with the highest IED counts in each session. Seizure frequency is expressed as the number of seizures per year. –: no history of spontaneous seizures. VS+ indicates that vestibular stimulation was applied during the experiment. Abbreviations: EEG = electroencephalography; LT = left temporal; RT = right temporal; LF = left frontal; RF = right frontal; LO = left occipital; RO = right occipital; LC = left central cortex; RC = right central cortex.

### Experimental procedures, analyses, and evaluation methods

**Figure 1:**
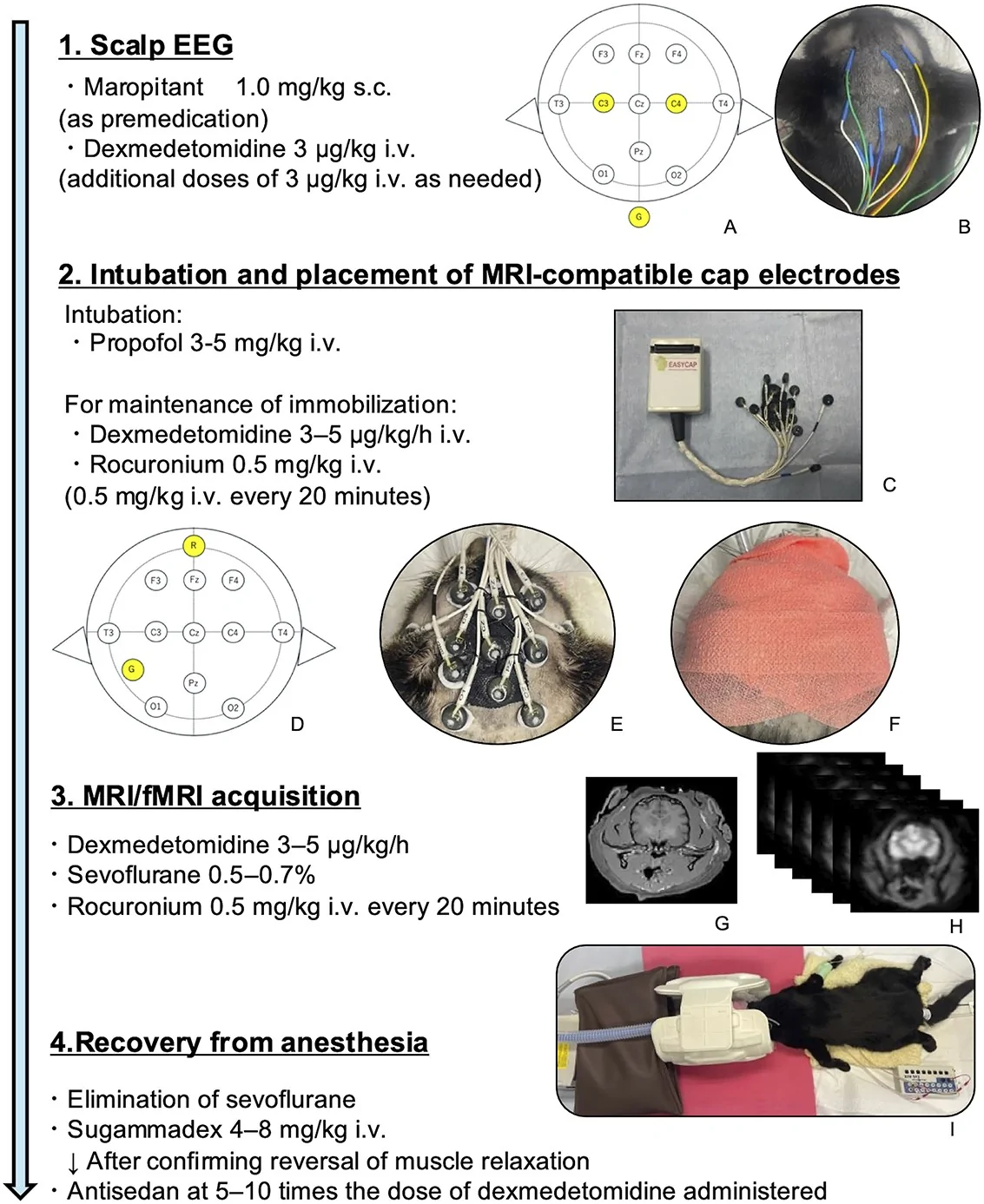
Experimental procedures and immobilization protocol. (A, B) Setup of the out-of-scanner scalp EEG recording. (C–F) MRI-compatible cap electrodes for cats and their placement. (G) Representative T1-weighted image and (H) echo planar imaging (EPI) acquired in the present study. (I) Overview of simultaneous EEG–fMRI acquisition. The cat’s head was positioned within a 16-channel flexible RF coil, and the connecting cables were secured with sandbags. Oxygen saturation, electrocardiography, and blood pressure were monitored during imaging.

**Figure 2:**
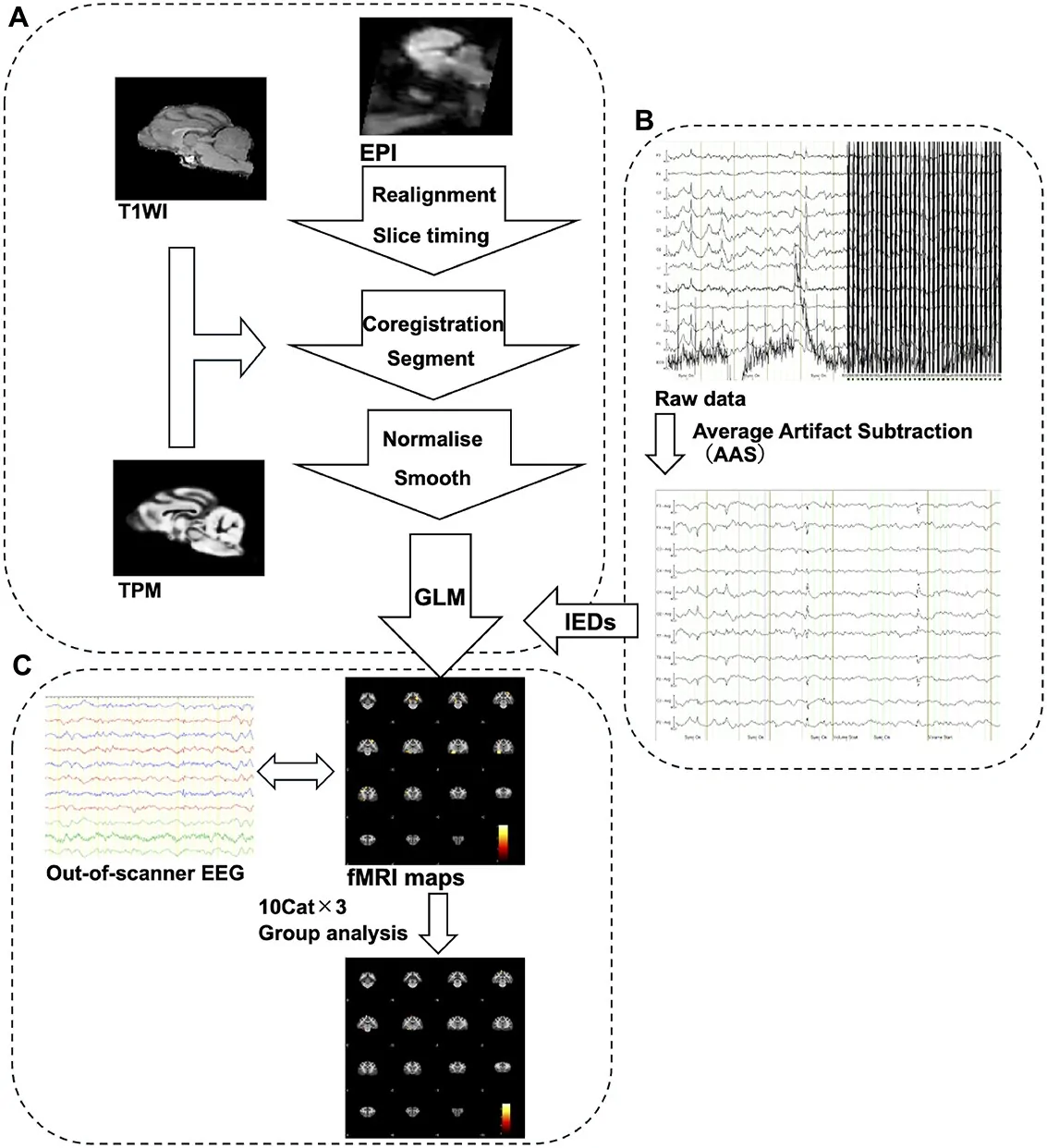
Flowchart of the preprocessing and analysis procedures used for generating BOLD activation images in this study. (A) fMRI preprocessing. (B) Artifact removal process for in-scanner EEG. (C) Generation and evaluation of BOLD activation images. Abbreviations: BOLD = blood oxygenation level dependent; T1WI = T1-weighted imaging; TPM = tissue probability map; EPI = echo planar imaging; AAS = average artifact subtraction; IEDs = interictal epileptiform discharges; GLM = general linear model.

Our exploratory pilot study was designed to develop and evaluate a methodological workflow of EEG-fMRI for future veterinary clinical application. Each cat underwent 3 experimental sessions, each consisting of scalp EEG performed outside the MRI scanner using a digital EEG system with subcutaneous needle electrodes (out-of-scanner EEG), placement of MRI-compatible cup electrodes, and simultaneous EEG and BOLD fMRI acquisition inside the MRI gantry (in-scanner EEG; Figure 1). Out-of-scanner EEG was performed under dexmedetomidine sedation, whereas EEG-fMRI was conducted under immobilization with additional rocuronium and low-dose sevoflurane, followed by reversal with atipamezole and sugammadex. The IEDs were manually counted in a 10-minute analyzable segment of the 20-25-minute EEG recording, and the 3 most active regions were identified for each session. For in-scanner EEG, MRI-compatible electrodes adapted for use in cats were connected to an MRI-compatible amplifier, using the same electrode components and amplifier as MRI-compatible EEG systems used in humans. After artifact removal, IED onsets were marked in BrainVision Analyzer 2 for fMRI analysis. The fMRI data were preprocessed and analyzed using SPM12 and Stolzberg’s feline template.^22^ The IEDs were modeled as event-related regressors convolved with the canonical hemodynamic response function (HRF) in a general linear model (GLM). The BOLD activation maps were evaluated sequentially using descending statistical thresholds. Maps were first assessed at a family-wise error-corrected threshold of *P* < .05 and an uncorrected threshold of *P* < .001. When activation was not consistently detected at these thresholds, a more liberal exploratory threshold was used to evaluate BOLD activation patterns (Figure 2).

**Figure 3:**
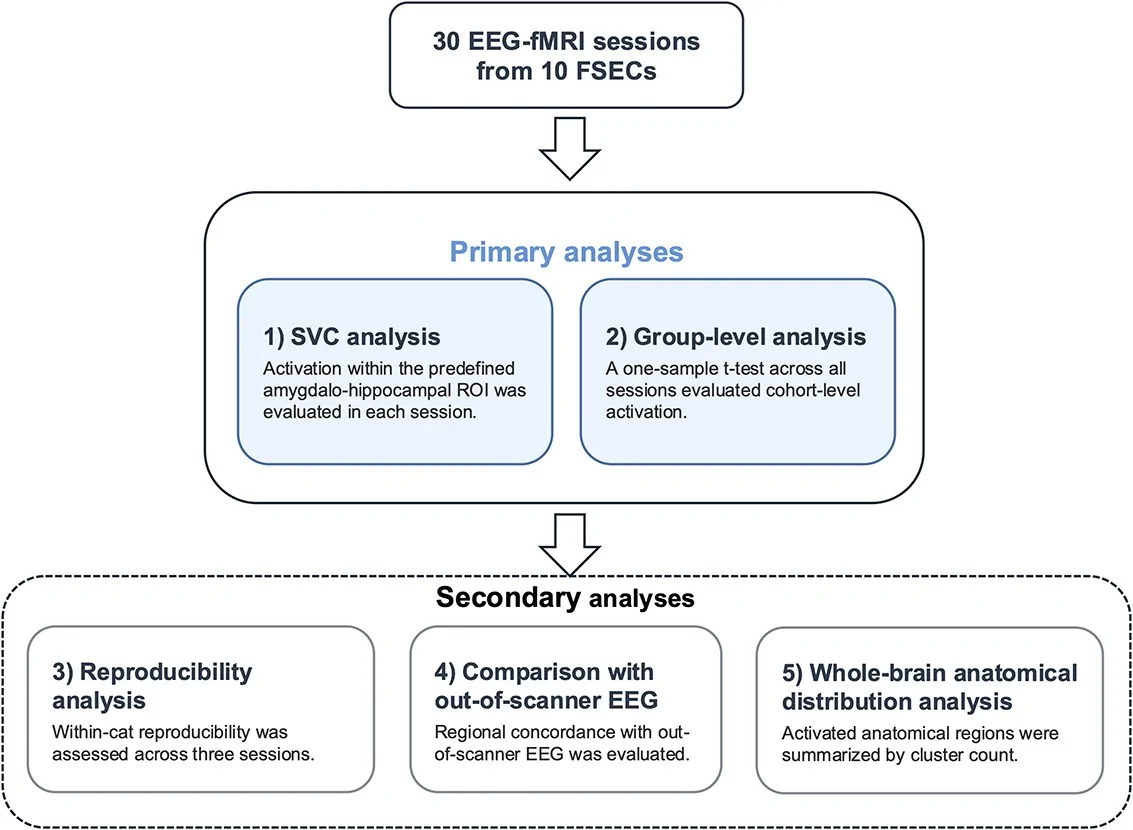
Overview of the evaluation methods used for the results in this study. Primary analyses consisted of small-volume correction (SVC) analysis within the predefined amygdalo-hippocampal ROI and group-level analysis across all sessions. Secondary analyses consisted of within-cat reproducibility analysis, comparison with out-of-scanner EEG, and whole-brain anatomical distribution analysis.

Evaluation consisted of primary and secondary analyses (Figure 3). Primary analyses assessed IED-related BOLD activation within the predefined amygdalo-hippocampal region using 2 approaches: session-level region of interest (ROI) analysis with small-volume correction (SVC) and cohort-level group analysis using a 1-sample *t-*test across all 30 sessions. Secondary analyses evaluated within-cat reproducibility across 3 sessions, regional concordance between out-of-scanner EEG and EEG-fMRI, and whole-brain anatomical distribution of activated clusters. For secondary analyses, activated regions were categorized into predefined anatomical groups, and the top 3 activated regions were selected for each session as described in the Appendix.

The 95% CIs for the observed proportions were calculated using the Wilson method in R.

Detailed methods are provided in the Appendix.

## Results

### Devices

In the our study, EEG-fMRI (ie, simultaneous EEG recording and BOLD fMRI acquisition) was technically completed in all 30 sessions using the modified MRI-compatible EEG electrodes and amplifier.

### Immobilization

In all cats, the immobilization protocol using dexmedetomidine, rocuronium, and low-dose sevoflurane allowed stable acquisition during in-scanner EEG and fMRI acquisition without apparent motion artifacts. Although some cats moved during electrode placement, additional sedative doses within the predefined range achieved sufficient immobilization during EEG-fMRI. The median immobilization time (from the first dexmedetomidine administration to completion of imaging) was 108 minutes (range, 82-124 minutes; Supplementary Table S1).

### Out-of-scanner EEG

Scalp EEG recordings were obtained under dexmedetomidine alone, as previously reported.^10^ The IED counts and the top 3 regions identified based on these counts are presented in Table 1.

### In-scanner EEG

The MRI-compatible EEG cup electrodes achieved impedances <10 kΩ in all cats, and no electrode displacement was observed during scanning. The median electrode placement time was 12 minutes (range, 5-26 minutes; Supplementary Table S1).

Gradient artifacts on in-scanner EEG were effectively removed using average artifact subtraction (AAS), with no substantial residual artifacts interfering with visual EEG interpretation. The EEG signals and IEDs could be visually assessed using average reference (AV) and bipolar montages. During the 12-minute analysis window, the median number of IEDs incorporated into the fMRI analysis was 71 (6 IEDs/minute; range, 38-127).

### fMRI Preprocessing

All fMRI datasets were successfully preprocessed using the same software and workflow as used in humans,^23^ allowing reliable visualization of BOLD responses mapped onto structural images.

### BOLD activation results

**Figure 4:**
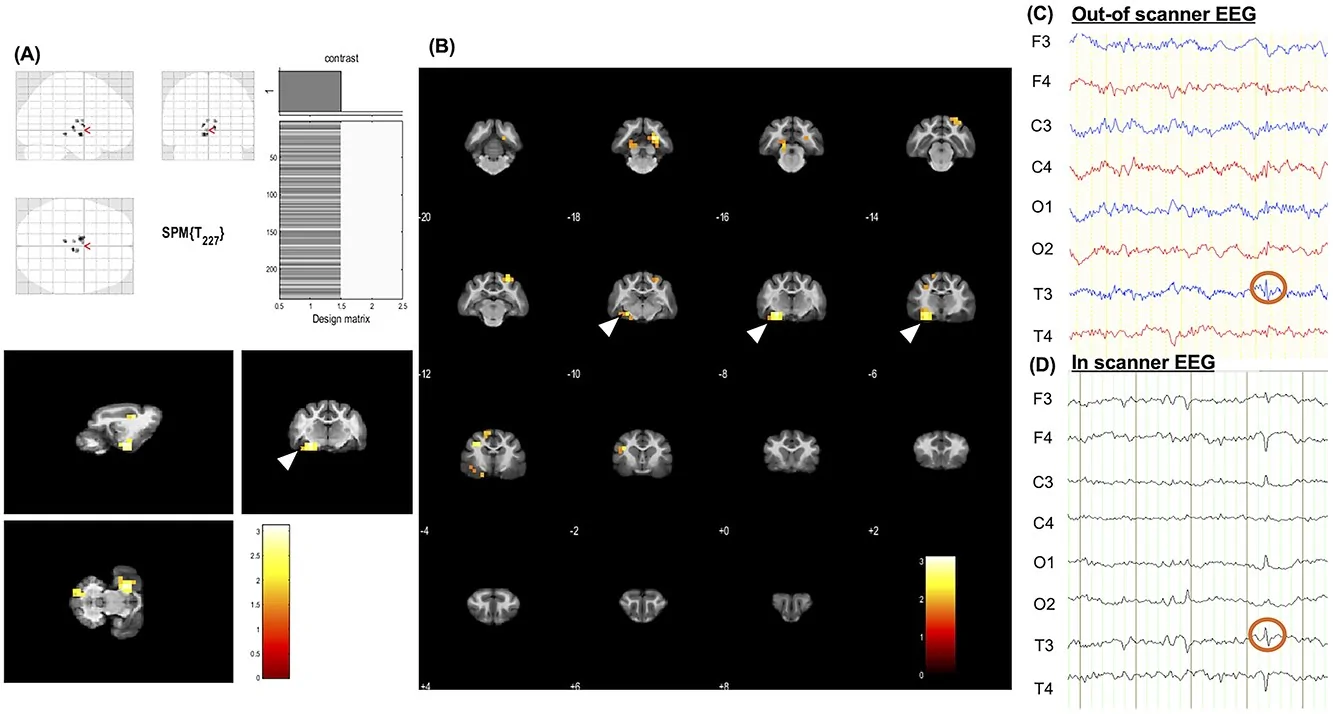
Example of the individual EEG-fMRI results (cat 4). Brain activation with a peak maximum in the left ventral hippocampus was observed in the first session of cat 4 (A, B; arrowheads indicate hippocampal activation), showing concordance with frequent left temporal spikes on scalp out-of-scanner (C) and in-scanner (D) EEGs.

The BOLD activation maps were first evaluated using more stringent statistical thresholds. No activation survived correction for multiple comparisons at a family-wise error-corrected *P* < .05. Activation was identified in 2/30 sessions at *P* < .001 (uncorrected) and in 19/30 sessions at *P* < .01 (uncorrected), both with a minimum cluster size of 3 voxels (k ≥ 3). At the exploratory threshold of *P* < .05 (uncorrected, k ≥ 3), BOLD activation was detected in all 30 sessions (Figure 4, Supplementary Table S2). Accordingly, subsequent analyses in our pilot study were performed using this exploratory threshold.

### Primary analyses

#### Small-volume correction analysis

At the exploratory threshold, activation within the predefined amygdalo-hippocampal ROI was detected in 16/30 sessions (53.3%; 95% CI, 35.4%-70.6%). At the individual level, 5/10 cats (50.0%; 95% CI, 23.7%-76.3%) showed amygdalo-hippocampal activation in at least 2 of 3 sessions.

#### Group-level analysis

**Figure 5:**
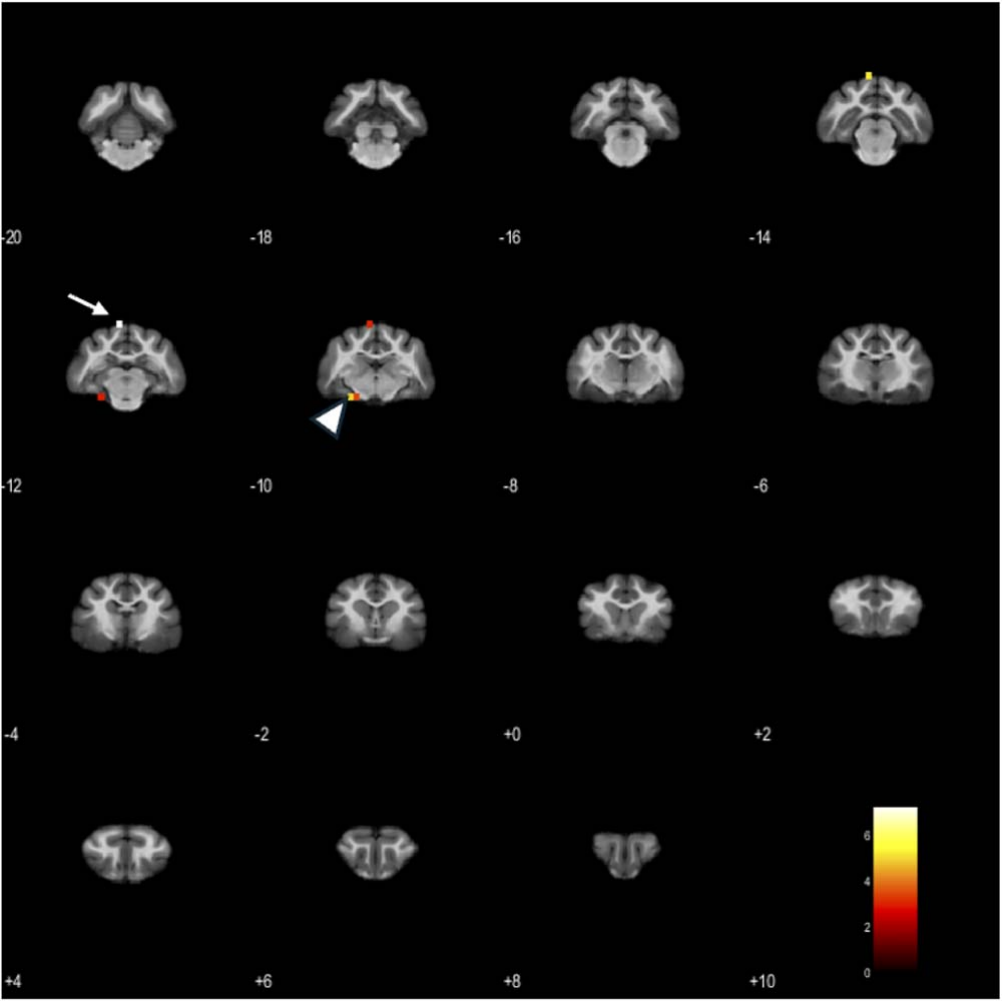
BOLD activation maps from the group-level analysis. **Group-level analysis based on all sessions (*n* = 30) from 10 cats.** Regions identified at ***P* < .01 (uncorrected, k ≥ 3) are shown**. Further details identified at ***P* < .05 (uncorrected, k ≥ 3)** are provided in the Supplementary Table S3. The peak maximum was located in the **left marginal gyrus of the parietal cortex** (arrow), and the largest cluster was observed extending from the **left parahippocampal gyrus to the left hippocampal body** (arrowhead).

No activation cluster survived correction for multiple comparisons at a family-wise error-corrected threshold of *P* < .05 in the group-level analysis. The largest activation cluster extended from the left parahippocampal gyrus to the hippocampal body (*P* < .01 uncorrected, k ≥ 3; peak *P* < .001 uncorrected; k = 17). The lowest *P*-value was located in the left marginal gyrus (*P* < .001 uncorrected, k = 7). When the amygdalo-hippocampal ROI was applied, a similar activation pattern was observed within the left parahippocampal–hippocampal region (Figure 5, Supplementary Table S3).

### Secondary analyses

Secondary analyses, including within-cat reproducibility, regional concordance between out-of-scanner EEG and EEG-fMRI, and whole-brain anatomical distribution, are summarized in Supplementary Tables S4 and S5. Briefly, reproducible activation was observed in 3/10 cats (30%; 95% CI, 10.8%-60.3%). Regional concordance between out-of-scanner EEG and EEG-fMRI was observed in 9/20 analyzable sessions (45%; 95% CI, 25.8%-65.8%), and 2/10 cats (20%; 95% CI, 5.7%-51.0%) showed both regional concordance and reproducible activation in at least 2 of 3 EEG-fMRI sessions. In the exploratory whole-brain anatomical analysis, activations included the hippocampus, parahippocampal gyrus, amygdala, piriform lobe, and other anatomically related regions.

## Discussion

To the best of our knowledge, ours is the first exploratory study to describe and evaluate EEG-fMRI using a familial epilepsy model in cats as an initial step toward future veterinary clinical application. Previous animal fMRI studies have been reported in dogs, cats, and non-human primates.^24–29^ However, animal EEG-fMRI investigations have been limited to experimental models in monkeys, sheep, mice, and rats, and were primarily designed for translational research relevant to human neuroscience and medicine.^30^ These models were not intended for veterinary clinical application, and therefore, their MRI coils, electrode systems, and immobilization or awake protocols may not reflect clinical conditions encountered in veterinary practice. In our study, EEG-fMRI was performed using a clinically realistic setup based on equipment available in routine veterinary practice and components of clinical EEG-fMRI systems used in humans. The MRI-compatible EEG electrodes used for in-scanner EEG were adapted for use in cats by modifying the electrode array placement and cable configuration, whereas the electrode components and MRI-compatible EEG amplifier were the same as those used in MRI-compatible EEG systems designed for use in humans. The MRI scanner and radiofrequency (RF) coil also were standard clinical devices. Using this setup, simultaneous EEG recording and fMRI acquisition could be technically completed in all sessions.

Key methodological challenges addressed in this pilot study included adapting MRI-compatible EEG electrodes for the head size of cats, developing an immobilization protocol that allowed stable image acquisition and interpretable EEG recordings, selecting an fMRI analysis workflow applicable to cats, and assessing whether IED-related BOLD responses could be visualized at an exploratory threshold under these conditions.

The electrode system consisted of 13 electrodes, including 11 recording electrodes, 1 ground electrode, and 1 reference electrode. Because head size in cats varies less markedly than in dogs, this electrode arrangement may be applicable to a broad range of cats. In contrast, for future studies in dogs, substantial variation in head size and shape among breeds will require adjustments in the number and placement of electrodes as well as cable length.

The immobilization protocol used in our study allowed stable EEG acquisition without apparent movement during MRI scanning. Although no established consensus exists, medetomidine or dexmedetomidine is commonly used for scalp EEG recording in veterinary medicine.^10^^,^^31^ However, animals sedated with these agents may remain responsive to loud auditory stimuli during MRI scanning.^32^ In our preliminary testing using dexmedetomidine continuous rate infusion (CRI) alone, the cats were likely aroused by MRI acoustic noise, resulting in movement during scanning and necessitating termination of the acquisition. Therefore, we combined the muscle relaxant, rocuronium, with low-dose sevoflurane to achieve movement-free immobilization. Rocuronium requires endotracheal intubation but has been shown not to affect EEG or cerebral blood flow in dogs, supporting its use as part of an immobilization protocol for EEG-fMRI.^33^ Sevoflurane can prolong muscle relaxation but may induce EEG abnormalities at high concentrations.^34^^,^^35^ However, no obvious EEG changes were observed at the low doses used in our study. Although awake fMRI has been achieved in primates, rodents, and trained dogs, such approaches are not practical for clinical veterinary patients because of facility limitations, required training, and variability in temperament. Therefore, immobilization protocols similar to the present approach may provide a practical framework for clinical EEG-fMRI studies in dogs and cats.

Electroencephalographic recording suitable for visual identification of IEDs and subsequent analysis was obtained in all cats. Careful clipping and degreasing, followed by electrode paste application, enabled impedance values ≤10 kΩ in every session. Although EEG-fMRI in humans typically targets ≤5 kΩ, achieving such low impedance is often difficult in animals because of thick skin and dense hair coats.^36^ Therefore, setting a practical impedance target of ≤10 kΩ is reasonable for veterinary applications. Although some breeds may not require clipping, the thickness of the undercoat and the presence of skin oils often necessitate hair removal to ensure reliable data acquisition. In cases evaluated with epilepsy surgery in mind, shaving is generally acceptable, but shaving performed solely for EEG–fMRI examinations may be cosmetically undesirable, making owner consent more difficult to obtain.

Four main types of artifacts are known to occur during EEG acquisition inside the MRI scanner: gradient artifacts generated by magnetic field gradients,^37^ motion artifacts caused by movement and associated induced currents,^38^ ballistocardiographic artifacts arising from cardiac pulsation and blood flow,^39^ and environmental artifacts produced by scanner room noise and vibration.^40^ The AAS method used in our study is a standard artifact removal technique used in humans for EEG-fMRI and produced interpretable EEG signals in our dataset as well.^37^ Appropriate immobilization, short cable lengths, and securing cables with sandbags further minimized artifact contamination.^41^ Ballistocardiographic artifacts also were negligible, as confirmed by simultaneous evaluation of ECG and EEG. However, interpretation of artifact-corrected EEG remains challenging even in humans, including for experienced readers.^13^ Therefore, additional artifact removal approaches, such as independent component analysis,^42^ principal component analysis,^43^ canonical correlation analysis,^44^ and optimal basis sets,^45^ should be considered to further improve EEG quality in future studies.

In our study, IED (spike) detection was performed visually. Although visual marking is also standard in humans,^13^ automated approaches using independent component analysis or deep learning have been increasingly reported, and future work should incorporate these methods.^46–48^ In our study, the median IED count during the 12-minute scan was 71 spikes. In humans, approximately 93 spikes over a 60-minute scan (1.6 spikes/minute; range, 10-1132) are typically required to obtain clear BOLD responses.^49^ Despite the higher spike rate observed in our cats, no BOLD activation survived higher statistical thresholds and correction for multiple comparisons. This result may have been a consequence of multiple factors, including limitations in spike extraction, partial volume effects, and other physiological constraints, as discussed later. In our study, all IEDs observed during in-scanner EEG were extracted and used for fMRI analysis. However, future comparisons with out-of-scanner EEG, especially when isolating specific spike types, will require consistent immobilization conditions across recordings. In our study, out-of-scanner EEG was recorded under dexmedetomidine sedation alone, whereas in-scanner EEG was recorded under a combination of dexmedetomidine, rocuronium, and low-dose sevoflurane.

The fMRI acquisition under the selected parameters allowed analyzable activation maps to be generated. No noticeable artifacts from EEG electrodes or cables were observed, consistent with reports in humans indicating that such artifacts generally are negligible.^50^ Our study was designed for clinical application, and therefore we used the standard 3 T MRI system and coil commonly employed in veterinary practice. In contrast, fMRI studies in rodents and primates often use dedicated coils and higher field strengths to maximize signal-to-noise ratio (SNR), which represents a limitation of our clinically oriented model. However, small animal-specific coils, such as strap-type coils now offered by some manufacturers, may further improve SNR in future studies.

The scan duration in our study was 12 minutes, shorter than the 30-60 minutes typically used in humans for EEG-fMRI.^41^ This duration reflects the standard single-session fMRI acquisition time for the MRI system used in our study. Although shorter scans decrease anesthetic and immobilization stress, extending acquisition time in clinical cases where scanning opportunities are limited would allow detection of more IEDs and potentially yield clearer activation. Given the stable immobilization achieved in our study, future studies may consider extending scan duration to 20-30 minutes using the same protocol.

Image preprocessing and visualization were performed using a brain template for cats previously employed in several MRI/fMRI studies.^22^ Cats exhibit relatively little inter-individual variation in brain morphology; therefore, this template provides sufficient anatomical accuracy for fMRI analysis. In contrast, dogs show considerable breed- and size-related variation in brain structure, and even individuals within the same breed can differ substantially. Although several dog templates have been reported, no high-precision template that accommodates all breeds or skull types is currently available.^51–53^ Therefore, future EEG-fMRI studies in dogs will likely require the creation of individualized or breed-specific templates.

Regarding spatial smoothing in the preprocessing, studies in humans commonly use a full width at half maximum (FWHM) of 8 mm, whereas veterinary fMRI studies typically apply 2-4 mm depending on voxel size.^26^^,^^27^^,^^29^ In our study, we used an FWHM of 4 mm to facilitate clearer visualization of cluster size, but an FWHM of 2 mm may be more appropriate for finer-grained analyses.

The primary analyses provided cohort-level findings of IED-related BOLD activation within the predefined amygdalo-hippocampal region of FSECs. The SVC analysis detected activation within the predefined amygdalo-hippocampal ROI in 16/30 sessions, and group-level analysis identified the largest activation cluster extending from the left parahippocampal gyrus to the hippocampal body. These findings are consistent with previous reports of amygdalo-hippocampal involvement in FSECs based on scalp EEG, structural MRI, intracranial EEG, and histopathology.^12^^,^^19–21^ Therefore, our results support the use of EEG-fMRI as a hypothesis-generating method for exploring IED-related network activity in this familial feline model.

In contrast, the secondary analyses showed limited within-cat reproducibility and regional concordance with out-of-scanner EEG. Reproducible BOLD activation was observed in 3/10 cats, and regional concordance between out-of-scanner EEG and EEG-fMRI was observed in 9/20 analyzable sessions. These values were lower than those reported in EEG-fMRI studies in humans: a previous study^54^ reported reproducible findings in 5/8 patients who underwent repeated EEG-fMRI examinations at 3 T, whereas another study^55^ reported concordant BOLD responses in 29/33 patients with active EEG and contributory responses in 21/33 patients. These findings indicate substantial inter-session variability and suggest that, under the present protocol, EEG-fMRI is not yet sufficient as a standalone method for individual-level EZ localization. Future studies should improve spike classification, harmonize immobilization conditions between EEG recordings, and focus on clinically confirmed drug-resistant epilepsy cases to better evaluate individual-level reliability. The FSECs have been reported as a familial feline model showing temporal lobe epilepsy-like features, including limbic seizure semiology and amygdalo-hippocampal abnormalities.^12^^,^^19–21^ Although the primary analyses suggested amygdalo-hippocampal involvement at the cohort level, some sessions showed BOLD activation outside the amygdalo-hippocampal region or discordance between scalp EEG and EEG-fMRI. In humans with epilepsy, EEG-fMRI BOLD responses can extend beyond the presumed EZ and may represent propagation pathways or broader epileptic networks, rather than only the primary epileptogenic region.^13^^,^^56^^,^^57^ Therefore, such discordant or extra-amygdalo-hippocampal activations do not necessarily indicate simple false-positive findings, but also may reflect spatially distributed epileptic network activity that cannot be captured by scalp EEG alone. However, because intracranial EEG or surgical validation was not performed in our study, these possibilities remain speculative, and the observed activations cannot be interpreted as definitive localization of the EZ. Future studies should evaluate whether EEG-fMRI activation corresponds to intracranial EEG findings or surgical outcomes in cats with drug-resistant epilepsy undergoing epilepsy surgery, as reported in studies of humans.^58^^,^^59^

A major limitation of our study was the use of an uncorrected statistical threshold for BOLD activation. No activation survived correction for multiple comparisons. Therefore, our findings should be interpreted as exploratory and hypothesis-generating. This lack of corrected significance likely reflects both limited statistical power and decreased BOLD sensitivity under the present protocol. Potential contributing factors include small sample size, short acquisition time, use of clinical rather than dedicated small animal MRI hardware, and sedation- or immobilization-related attenuation of neurovascular coupling and vascular reactivity. Additional factors may include spatial resolution- and analysis-related limitations arising from the use of clinical MRI acquisition settings in small feline brains, including relatively large voxel dimensions compared with feline brain structures and increased partial-volume effects. Other possible contributors include suboptimal acquisition and preprocessing parameters for veterinary EEG-fMRI, potential mismatch between the canonical HRF derived from humans and the BOLD response of cats, and limited physiological noise correction. Future studies should optimize acquisition and analysis parameters, including scan duration, receiver coils, spatial smoothing, physiological noise correction, species-specific hemodynamic modeling, and the establishment of statistical thresholds appropriate for veterinary EEG-fMRI. Validation in clinically confirmed epilepsy surgery candidates using intracranial EEG, multimodal presurgical evaluations, and surgical outcomes also will be necessary.

Our study had some additional limitations related to its exploratory methodological design. First, FSECs were selected as a biologically relevant model for establishing EEG-fMRI methodology, but only a small number of cats were included, and several did not have documented clinical seizures. Therefore, the present cohort was not suitable for evaluating individual-level diagnostic accuracy. Second, the presumed amygdalo-hippocampal involvement was based on previous studies of this familial strain, but intracranial EEG or surgical confirmation was not performed in the cats included in our study. In addition, because the MRI-compatible cup electrodes used in our study were adapted from commercially available EEG electrodes designed for use in humans rather than newly developed specifically for cats, we did not perform additional technical validation, such as impedance comparisons with SC needle electrodes or power spectral density comparisons between electrode types. Third, out-of-scanner EEG and in-scanner EEG were recorded under different immobilization conditions, which may have affected spike characteristics and regional concordance. These limitations reflect the initial methodological nature of our study, and further evaluation in clinically confirmed drug-resistant epilepsy cases undergoing presurgical assessment is the next necessary step. Validation against intracranial EEG, multimodal imaging, and postoperative outcomes will be required to determine whether EEG-fMRI can contribute to clinical decision-making in veterinary epilepsy surgery. Once optimized and validated, EEG-fMRI also may provide a noninvasive framework for investigating epileptic networks, drug-related changes in brain activity, sleep-related EEG phenomena, and event-related brain responses in companion animals.^60–62^

## Conclusion and clinical importance

Our exploratory pilot study showed that simultaneous EEG-fMRI can be technically implemented in FSECs using a clinically realistic veterinary MRI setup. Cohort-level amygdalo-hippocampal activation was observed at an exploratory threshold, but individual-level reproducibility, EEG concordance, and statistical robustness remained limited. Further validation in clinical epilepsy surgery candidates, including comparison with intracranial EEG and surgical outcomes, is required before EEG-fMRI can be applied to clinical EZ localization.

## Supplementary Material

Supplementary_material_aalag209

## Data Availability

The raw data supporting the conclusions of this article will be made available by the corresponding author on request.

## Off-label antimicrobial declaration

Authors declare no off-label use of antimicrobials.

## Institutional animal care and use committee or other approval declaration

Approved by the Institutional Animal Care and Use Committee of Nippon Veterinary and Life Science University (approval numbers: 2023S-2, 2023 K-3, S2023K-3, 2024 K-2, S2024K-2, 2024 K-3; principal investigator: D.H.).

## Appendix

### Preliminary experiment

Before the study, both scalp (subcutaneous) needle electrodes for EEG and MRI-compatible EEG cap electrodes were placed simultaneously on the scalp of the same cats (*n* = 17), and it was confirmed under dexmedetomidine sedation that the MRI-compatible cup electrodes yielded EEG findings comparable to those obtained with needle electrodes.

Because it was difficult to maintain sufficient immobilization during MRI scanning with dexmedetomidine alone, the immobilization protocol was modified to include the combined use of rocuronium and inhaled sevoflurane. As a preliminary step, EEG recordings were sequentially obtained outside the scanner under 3 conditions: dexmedetomidine alone, dexmedetomidine combined with rocuronium, and dexmedetomidine combined with rocuronium and low-dose sevoflurane, and the resulting EEG findings were compared. As a result, a decrease in electromyographic activity and clearer spike delineation were observed after the administration of rocuronium, whereas the addition of sevoflurane did not induce any marked changes in EEG findings.

The effects of rocuronium were considered to result from the elimination of temporal muscle interference through muscle relaxation. Although neuromuscular blocking agents suppress not only body movement but also spontaneous respiration, thereby necessitating endotracheal intubation, previous studies in dogs have reported that rocuronium does not affect EEG activity or cerebral blood flow.^33^ Therefore, its use was considered essential for achieving sufficient immobilization with minimal impact on EEG analysis and cerebral hemodynamics during fMRI acquisition.

With respect to sevoflurane, although high concentrations have been reported to induce EEG abnormalities, no marked abnormal EEG findings were observed in our study because only low concentrations were used.^34^ In addition, sevoflurane is known to prolong the effects of neuromuscular blocking agents, thereby contributing to improved immobilization.^35^

Based on these preliminary experiments and considerations, out-of-scanner EEG was recorded under dexmedetomidine sedation alone, whereas in-scanner EEG was obtained under combined immobilization with dexmedetomidine, rocuronium, and sevoflurane in the study. Each dose is described below.

### Experimental procedures

#### Animals

Ten cats from an FSEC colony showing clear IEDs on scalp EEG were included in our exploratory pilot study, regardless of whether documented spontaneous clinical seizures were present. Because EEG-fMRI analyses require detectable IEDs as event-related regressors and a biologically relevant reference framework for interpretation of the resulting BOLD activations, FSECs were selected as an exploratory model for methodological evaluation. Previous studies using scalp EEG, structural MRI, intracranial EEG, and histopathology have suggested involvement of the amygdalo-hippocampal region in epileptogenesis in this familial strain^12^^,^^19–21^ Cats without documented clinical seizures were regarded as having presumed epileptic susceptibility based on their familial background and EEG findings, rather than individually confirmed clinical epilepsy. The cohort consisted of 5 males and 5 females, with a median age of 4.6 years (range, 1-12 years) and a median body weight of 3.5 kg (range, 2.1-5.3 kg). All cats underwent diagnostic evaluations equivalent to Tier III level of the International Veterinary Epilepsy Task Force criteria for idiopathic epilepsy in dogs, including MRI, cerebrospinal fluid analysis, and scalp EEG (Table 1  **in the main text**).

### Experimental procedures and immobilization protocol

Each cat underwent 3 sessions consisting of scalp EEG (out-of-scanner EEG), placement of MRI-compatible cap electrodes, and simultaneous EEG and BOLD-fMRI acquisition in the MRI gantry (In-scanner EEG; Figure 1  **in the main text**).

Before out-of-scanner EEG, maropitant (1 mg/kg IV; Cerenia, Zoetis, Japan) was administered as premedication, followed by dexmedetomidine sedation (3-5 μg/kg IV; Precedex, Pfizer, United States).

After completion of out-of-scanner EEG, endotracheal intubation was performed using propofol (3-5 mg/kg IV; Propofol, Toyama Chemical, Japan). During placement of MRI-compatible electrodes, anesthesia was maintained with a continuous rate infusion (CRI) of dexmedetomidine (3-5 μg/kg/h) and intermittent administration of rocuronium bromide (0.5 mg/kg IV every 20-25 minutes; Rocuronium, Maruishi Pharmaceutical, Japan).

During EEG-fMRI acquisition, heart rate, blood pressure, and oxygen saturation were continuously monitored. Anesthesia was maintained with a CRI of dexmedetomidine (3-5 μg/kg/h) and inhalation of sevoflurane (0.3%-0.5%; Sevoflo, Maruishi Pharmaceutical, Japan) in oxygen. Respiratory rate was mechanically controlled at 8-12 breaths/min, and lactated Ringer’s solution (3 mL/kg/h IV; Solulact, Terumo, Japan) was administered. Warming bags were used to maintain body temperature. When movement occurred during electrode placement or imaging, an additional dexmedetomidine bolus (1 μg/kg IV) was administered or the rocuronium dosing interval was shortened or both.

After imaging, atipamezole (30-50 μg/kg IV; Antisedan, Nippon Zenyaku Kogyo, Japan) and sugammadex sodium (4-8 mg/kg IV; Sugammadex, Nipro, Japan) were administered according to the final dexmedetomidine and rocuronium doses. The procedure was completed after confirmation of spontaneous respiration, stable vital parameters, and full recovery of consciousness.

### Out-of-scanner EEG

Out-of-scanner EEG was obtained using a digital electroencephalograph (Neurofax EEG-1200, Nihon Kohden, Japan) and a multichannel electrode box (JE-120A/64 ch, Nihon Kohden, Japan). After administration of dexmedetomidine, cats were positioned in sternal recumbency, and subcutaneous needle electrodes were used for recording. Recording electrodes were placed bilaterally over the frontal, central, temporal, and occipital regions, as well as at the midline sites F3 (left frontal), F4 (right frontal), C3 (left central), C4 (right central), T3 (left temporal), T4 (right temporal), O1 (left occipital), and O2 (right occipital) Fz (midline frontal), Cz (midline central), and Pz (midline central).^10^ An AV montage, calculated from all recording electrodes, was used for real-time observation.

The EEG was recorded with the following parameters: sampling frequency, 2000 Hz; high-cut filter, 60 Hz; time constant, 0.1-0.3 seconds; sensitivity, 5-10 μV/mm; notch filter, on; ground electrode, subcutaneous cervical region; system reference, C3 and C4; and a 15-second display window. During EEG acquisition, electrocardiography and respiratory rate were continuously monitored, and recordings were obtained for 20-25 minutes under immobilization.

### Placement of MRI-compatible cup electrodes

After completion of the out-of-scanner EEG measurement, the scalp was shaved and degreased with isopropanol to achieve optimal electrode impedance. The MRI-compatible EEG cup electrodes for use in cats were adapted from a commercially available human MRI-compatible EEG cap system (EASYCAP, Brain Products, Germany). For use in cats, flexible electrode arrays were used instead of the cap configuration used in humans, and the number of electrodes, electrode placement, and cable configuration were modified. The electrodes were applied using EEG electrode paste (Elefix V, Nihon Kohden, Japan) at the following sites: F3, F4, C3, C4, T3, T4, O1, O2, Fz, Cz, and Pz. Electrocardiography electrodes also were placed at the bilateral axillary regions and the umbilical area.

The EASYCAP electrodes were connected to an MRI-compatible EEG amplifier (BrainAmp MR, Brain Products, Germany), which was linked to a personal computer running EEG recording software (BrainVision, Brain Products, Germany). Electrode impedance was confirmed to be ≤10 kΩ for all electrodes before recording. To enhance adhesion, the cup electrodes were secured to the head using an adhesive bandage, and earplugs were applied for noise protection during MRI acquisition.

### E‌EG-fMRI acquisition

Magnetic resonance imaging was performed using a 3.0-T scanner (Vantage Centurian ZGO, Canon Medical Systems, Japan), with a 16-channel flexible RF coil (medium size). To minimize artifact contamination, a short cable (length, 10 cm) between the scalp electrodes and the EEG amplifier was used and fixed in a straight line with sandbags (Figure 1-I in the main text).

After final head positioning, a 3D T1-weighted image was acquired (3D- fast field echo, FFE; TR = 6.2 ms, TE = 2.9 ms, TI = 900 ms, FOV = 16 × 16 cm, slice thickness = 0.7 mm, matrix = 240 × 240, NEX = 1; acquisition time = 3 minutes 21 seconds). Subsequently, fMRI (2D- echo-planar imaging, EPI; TR = 3000 ms, TE = 25 ms, FOV = 15 × 15 cm, number of slices = 21, slice thickness = 3 mm, slice gap = 0.3 mm, matrix = 96 × 96; acquisition time = 12 minutes) and simultaneous in-scanner EEG recordings were performed.

In-scanner EEG recording was initiated immediately before fMRI acquisition using BrainVision Recorder. During scanning, EEG signals were monitored in real-time using an AV derivation with software capable of artifact reduction (BrainVision RecView, Brain Products, Germany). Body motion and vital signs were assessed by visual inspection of the EEG and ECG signals.

### Data analysis

#### Out-of-scanner EEG analysis

For out-of-scanner EEG, interictal discharges (IEDs), including spikes, polyspikes, spike-and-wave complexes, and sharp waves, were manually counted using referential, AV, and bipolar montages over a 10-minute analyzable segment. IEDs were categorized according to electrode location: F3, F4, C3, C4, T3, T4, O1, and O2. Based on IED counts, the top 3 regions with the highest IED frequency were selected for subsequent comparison with fMRI-derived activation sites. Midline electrodes (Fz, Cz, and Pz) were excluded from these analyses to facilitate comparison with fMRI activation patterns.

### In-scanner EEG analysis

In-scanner EEG during fMRI acquisition was processed using BrainVision Analyzer 2 (Brain Products, Germany). Gradient artifacts were removed using the AAS method. Artifact-corrected EEG signals were reviewed to confirm clear visualization in referential, AV, and bipolar montages.^37^ Processing parameters included a sampling rate of 500 Hz, a band-pass filter of 0.5-70 Hz, sensitivity of 3-5 μV/mm, AV montage, and a 15-seconds display window. All IEDs were identified manually, regardless of laterality or location, and a marker was placed at each IED onset (Figure 2  **in the main text**). For comparison with out-of-scanner EEG, the intended recording electrode locations were matched between out-of-scanner and in-scanner EEG using the same anatomical electrode labels. However, the reference electrode configuration differed because of the specifications of the MRI-compatible EEG system. Minor differences in electrode position were expected because of individual variation in head size and shape, as well as practical limitations of clinical electrode placement. Because the comparison was performed at the regional level rather than as point-by-point electrode localization, such minor variation was considered acceptable for our exploratory analysis.

### fMRI data preprocessing

Preprocessing and analysis of fMRI data were performed using MATLAB R2021b (MathWorks, Natick, MA, United States) and Statistical Parametric Mapping (SPM12, University College London, United Kingdom), following established preprocessing pipelines used in humans, the SPM12 manual, and previous fMRI studies in dogs and cats (Figure 2  **in the main text**).^23^^,^^29^ Digital imaging and communications in medicine (DICOM) images were converted to NIfTI format, aligned to the anterior and posterior commissures, realigned for motion correction, coregistered to structural images, segmented, and corrected for slice timing. Functional images were normalized and spatially smoothed.^22^ Structural 3D T1-weighted images were manually skull-stripped using MRIcroGL (Ver. 1.2.20220720, NITRC) to remove extracranial tissues before coregistration. Spatial smoothing was performed using a Gaussian kernel with a FWHM of 4 × 4 × 4 mm^3^.

### BOLD activation mapping

Interictal epileptiform discharges were modeled as event onsets and convolved with the canonical HRF to generate regressors for the GLM. Voxel-wise GLM analyses then were performed on the preprocessed images to obtain contrast maps, using the gray-matter probability maps from a previous study^22^ as masks. Contrast maps were visualized on the feline template,^22^ and activation additionally was verified on each cat’s normalized T1-weighted image.^22^ Because ours was an exploratory analysis, statistical significance was set at *P* (uncorrected) < .05 with a minimum cluster size (k) of ≥3 voxels. Cortical regions were defined using the previous template for cats,^22^ whereas subcortical structures were defined according to a previously published stereotaxic atlas.^63^ For comparison with out-of-scanner EEG, anatomical regions were categorized into predefined groups (Appendix Table A1). For each session, the region with the most significant *P*-value was designated Region 1, the region with the largest cluster was designated Region 2, and the region with the next most significant *P*-value region was designated Region 3.

**Table A1:** Definition of anatomical regions.

Brain area	Anatomical region
**F: Frontal**	anterior/posterior sigmoid gyrus, coronal gyrus, superior/inferior frontal gyrus, proreus gyrus, frontal cingulate gyrus, piriform lobe
**C: Central**	marginal gyrus, anterior suprasylvian gyrus, middle suprasylvian gyrus, middle ectosylvian, central cingulate gyrus
**T: Temporal**	anterior/middle/posterior sylvian gyrus, anterior ectosylvian gyrus, post ectosylvian gyrus, parahippocampal gyrus
**O: Occipital**	post suprasylvian gyrus, post marginal gyrus
**Deep gray matter**	hippocampal (T/O), amygdala (F/T)

The anatomical brain regions were defined based on a feline T1-weighted template image for the superficial gray matter and a feline brain atlas for the deep gray matter structures.^23^^,^^63^ These regions were then classified into 4 brain regions as shown in the Table. The hippocampus and amygdala, which are deep gray matter structures, were classified as either T or O and as either F or T, respectively.

### Evaluation of the Results

An overview of the evaluation workflow is presented in Figure 3  **in the main text**. Evaluation consisted of primary analyses and supplementary descriptive analyses.

#### Primary analyses

##### Small-volume correction analysis

Amygdalo-hippocampal regions of interest (ROIs) were manually created in MRIcroGL based on gray matter probability maps. Small-volume correction (SVC) analysis was performed to assess IED-related BOLD activation specifically within the predefined amygdalo-hippocampal ROIs. The presence or absence of activation within the ROI was recorded for each session.

### Group-level analysis

A group-level 1-sample *t-*test was performed using all 30 contrast maps to assess whether IED-related BOLD activation was detected in the amygdalo-hippocampal region at the FSEC cohort level. Activation was considered significant at an uncorrected threshold of *P* < .05 with ≥3 contiguous voxels (k ≥ 3), consistent with the individual level analyses.

### Secondary analyses

#### Reproducibility analysis

For each cat, reproducibility of EEG-fMRI activation was assessed across the 3 sessions. An anatomical region was considered reproducibly activated when it appeared among the top 3 activated regions in at least 2 sessions.

### Comparison with out-of-scanner EEG

For each session, the top 3 regions with the highest IED frequency in out-of-scanner EEG were compared with the top 3 EEG-fMRI-activated regions. Concordance was defined as the presence of at least 2 matching regions.

### Whole-brain anatomical distribution analysis

Whole-brain anatomical distribution analysis was performed to describe the distribution of activated clusters across all sessions. Activated clusters were summarized by anatomical region based on cluster counts. This analysis was descriptive and was not used as a primary measure of localization accuracy.

### Statistical analysis

The 95% CIs for the observed proportions were calculated using the Wilson method in R (R Foundation for Statistical Computing, Vienna, Austria).

## Abbreviations

AAS: average artifact subtraction
AV: average reference
BOLD: blood-oxygenation-level-dependent
ECoG: electrocorticography
EEG: electroencephalography
EEG-fMRI: electroencephalography–functional magnetic resonance imaging
EZ: epileptogenic zone
FDG-PET: fluorodeoxyglucose positron emission tomography
FSECs: familial spontaneous epileptic cats
FWHM: full width at half maximum
fMRI: functional magnetic resonance imaging
IEDs: interictal epileptiform discharges
MRI: magnetic resonance imaging
ROI: region of interest
SEEG: stereo-electroencephalography
SNR: signal-to-noise ratio
SVC: small volume correction
VEEG: video-electroencephalography (electroencephalography with video monitoring)
