# Supplementary material for: Electroencephalography-functional magnetic resonance imaging using a familial temporal lobe epilepsy model in cats: an exploratory methodological pilot study
Source: J Vet Intern Med. 2026 Sep 21;40(5):aalag209. doi: 10.1093/jvimsj/aalag209 (PMC13591485; doi:10.1093/jvimsj/aalag209)
Supplement: Supplementary file 1 [file supplementary_material_aalag209.zip › Supplementary_data_DH-clean_for_publishing_aalag209.docx]

| **Cat No.** | **Session #** | **Electrode placement time（minutes）** | **Total immobilization time (minutes)** |
| --- | --- | --- | --- |
| **Cat1** | 1 | 9 | 119 |
|  | 2 | 14 | 119 |
|  | 3 | 10 | 110 |
| **Cat2** | 1 | 26 | 120 |
|  | 2 | 10 | 106 |
|  | 3 | 17 | 99 |
| **Cat3** | 1 | 23 | 110 |
|  | 2 | 12 | 103 |
|  | 3 | 5 | 108 |
| **Cat4** | 1 | 9 | 117 |
|  | 2 | 7 | 103 |
|  | 3 | 12 | 89 |
| **Cat5** | 1 | 10 | 103 |
|  | 2 | 17 | 124 |
|  | 3 | 20 | 110 |
| **Cat6** | 1 | 7 | 100 |
|  | 2 | 10 | 93 |
|  | 3 | 12 | 122 |
| **Cat7** | 1 | 18 | 127 |
|  | 2 | 8 | 121 |
|  | 3 | 7 | 82 |
| **Cat8** | 1 | 10 | 106 |
|  | 2 | 17 | 108 |
|  | 3 | 20 | 119 |
| **Cat9** | 1 | 10 | 128 |
|  | 2 | 12 | 108 |
|  | 3 | 10 | 92 |
| **Cat10** | 1 | 12 | 104 |
|  | 2 | 20 | 97 |
|  | 3 | 13 | 112 |
| **Median time** |  | 12 (5-26) | 108 (82-124) |

**Supplementary Table S1. Duration of immobilization for each procedure**

The time required for electrode placement and the total immobilization time were recorded for all sessions. The total immobilization time was defined as the duration from dexmedetomidine administration to the completion of the examination.

**Supplementary Table S2 (Separate file). Results of BOLD activation across all sessions.**

Source data were compiled and summarized using Microsoft Excel (each sheet corresponds to an individual cat). Blank cells indicate additional local maxima within the same cluster (cluster size k shown for the first peak only).

**Supplementary Table S3. BOLD activation details from the group-level analysis**

| **Localization** | **cluster size (k)**  **(>3)** | **peak Z**  **(>1.9)** | **peak level**  **(<0.05 uncorrect)** |
| --- | --- | --- | --- |
| **Left marginal gyrus** | 7 | 4.1 | <0.001 |
| **Left parahippocampal gyrus** | 17 | 3.4 | <0.001 |
| **Left Formatio hippocampalis ventralis** | 17 | 2.2 | 0.01 |
| **Right cingulate gyrus** | 6 | 2.6 | 0.004 |
| **Right Formatio hippocampalis ventralis** | 3 | 2.4 | 0.009 |
| **Right middle ectosylvian gyrus** | 3 | 2.2 | 0.01 |
| **Mammillary body** | 9 | 2.2 | 0.01 |
| **Right cingulate gyrus** | 6 | 2.2 | 0.02 |
| **Left olfactory bulb** | 4 | 2.1 | 0.02 |
| **Left ectosylvian gyrus** | 7 | 2 | 0.02 |

Results obtained using more lenient statistical thresholds than those applied in Figure 5 of the main text are shown. These regions were identified using a threshold of ***p* < 0.05 (uncorrected, k ≥ 3).** Under these conditions, a greater number of activation clusters were detected, predominantly in regions associated with the limbic system.

**Supplementary Table S4 (Separate file). Results of Comparison between out-of-scanner EEG and BOLD activation regions**

This table summarizes the secondary analyses, including within-cat reproducibility of EEG-fMRI activation and regional concordance between out-of-scanner EEG and BOLD activation. Reproducibility was defined as detection of the same anatomical region among the top three EEG-fMRI activation regions in at least two of the three sessions for each cat. Reproducible activation was observed in 3/10 cats: Cat 1, Cat 4, and Cat 10. Also, anatomical regions showing reproducible activation in at least two of the three trials are indicated in red.

For comparison with out-of-scanner EEG, the top three regions determined by IED frequency on scalp EEG were compared with the top three regions selected based on BOLD activation. After exclusion of sessions unsuitable for regional comparison, 20/30 sessions were included. Regional concordance, defined as at least two matching regions, was observed in 9/20 sessions. Among these concordant sessions, amygdalo-hippocampal activation was detected in 6/9 sessions and in 6/30 sessions overall. Cats showing concordance with scalp EEG are indicated by ♭, and cats showing anatomical reproducibility are indicated by ✱. Source data were compiled and summarized using Microsoft Excel.

**Supplementary Table S5. Summary of anatomical regions showing BOLD activation in all sessions (Whole-Brain analysis)**

| **Region category** | **Brain region** | **Activation frequency (95% CI)** |
| --- | --- | --- |
| **Cerebral cortex** | Olfactory bulb | 3.0% (1.4-6.5) |
|  | Frontal lobe | 5.1% (2.8-9.1) |
|  | Temporal lobe | 6.6% (3.9-11.0) |
|  | Parietal lobe | 14.2% (10.0-19.8) |
|  | Occipital lobe | 6.1% (3.5-10.3) |
| **Limbic system** | Hippocampus and parahippocampal gyrus | 14.7% (10.4-20.3) |
|  | Piriform lobe and amygdala | 6.6% (3.9-11.0) |
|  | Cingulate gyrus | 7.1% (4.3-11.6) |
|  | Thalamus | 3.6% (1.7-7.2) |
|  | Mammillary body | 2.0% (0.8-5.1) |
|  | Septum | 0.5% (0.1-2.8) |
| **Other area** | Caudate nucleus | 1.0% (0.3-3.6) |
|  | Cerebellum | 16.8% (12.2-22.6) |
|  | Midbrain | 3.0% (1.4-6.5) |
|  | Pons | 4.6% (2.4-8.5) |
|  | Medulla oblongata | 5.1% (2.8-9.1) |

The table and pie chart summarize the whole-brain anatomical distribution of activated clusters across all sessions. Activated clusters were summarized by anatomical region based on cluster counts, and the corresponding proportions with 95% confidence intervals are shown in the table. This analysis was descriptive and was not used as a primary measure of localization accuracy.

Activation within the hippocampus/parahippocampal gyrus and piriform lobe/amygdala accounted for 14.7% and 6.6% of activated clusters, respectively. Activated clusters were also distributed across cortical, subcortical, brainstem, and cerebellar regions.
