# Supplementary material for: Electroencephalography-functional magnetic resonance imaging using a familial temporal lobe epilepsy model in cats: an exploratory methodological pilot study
Source: J Vet Intern Med. 2026 Sep 21;40(5):aalag209. doi: 10.1093/jvimsj/aalag209 (PMC13591485; doi:10.1093/jvimsj/aalag209)
Supplement: Supplementary file 1 [file supplementary_material_aalag209.zip › Supplementary_material_aalag209.docx]

**Supplementary material:** The file named “Supplementary data” includes Supplementary Tables S1, S3, and S5, as well as the captions for Supplementary Tables S2 and S4. Data for Supplementary Tables S2 and S4 are provided as separate Excel files.
